# Is Neurodegenerative Disease a Long-Latency Response to Early-Life Genotoxin Exposure?

**DOI:** 10.3390/ijerph8103889

**Published:** 2011-09-29

**Authors:** Glen E. Kisby, Peter S. Spencer

**Affiliations:** 1 Department of Basic Medical Sciences, Western University of Health Sciences, College of Osteopathic Medicine of the Pacific Northwest, Lebanon, OR 97355, USA; E-Mail: gkisby@westernu.edu; 2 Global Health Center, Center for Research on Occupational & Environmental Toxicology, and School of Medicine Department of Neurology, Oregon Health & Science University, Portland, OR 97239, USA

**Keywords:** Guam, cycad, methylazoxymethanol (MAM), β-*N*-methylamino-L-alanine (L-BMAA), DNA damage, tauopathy, neurodegenerative disease, amyotrophic lateral sclerosis (ALS), parkinsonism-dementia, nitrosamines, formaldehyde

## Abstract

Western Pacific amyotrophic lateral sclerosis and parkinsonism-dementia complex, a disappearing neurodegenerative disease linked to use of the neurotoxic cycad plant for food and/or medicine, is intensively studied because the neuropathology (tauopathy) is similar to that of Alzheimer’s disease. Cycads contain neurotoxic and genotoxic principles, notably cycasin and methylazoxymethanol, the latter sharing chemical relations with nitrosamines, which are derived from nitrates and nitrites in preserved meats and fertilizers, and also used in the rubber and leather industries. This review includes new data that influence understanding of the neurobiological actions of cycad and related genotoxins and the putative mechanisms by which they might trigger neurodegenerative disease.

## 1. Introduction

Neurobiology provides a solid foundation on which to explain the molecular pathogenesis of most chemically triggered brain diseases, including self-limiting human neurodegenerative disorders. For example, substances with neurotoxic potential may bind to membrane receptors, enter cells via membrane channels, interfere with mitochondrial function, disrupt axonal transport, or perturb synaptic integrity. The resulting neurocellular dysfunction presages functional changes that may remain sub-clinical or find clinical expression in minutes, hours, days or weeks. The associated clinical disorder peaks over time, plateaus and either persists or resolves. Thus, chemical exposure produces a *self-limiting* neurological disorder by activating an abnormal physiologic state that reverses or, with cell loss, persists, but *does not advance*. True, the neurological condition may relapse or progress in the event of continued chemical exposure or the release of an endogenously stored substance, but chemicals that act through the foregoing cellular mechanisms are considered unable to trigger a self-propelling disorder that results in the relentless, unstoppable advance of neurological disease. If chemicals are ever found to induce *progressive* and ultimately fatal neurodegenerative diseases, a fundamentally different neurotoxic molecular mechanism must be involved [1,2].

Ten years ago, we embarked on a voyage to discover that mechanism because of the existence of an intensively studied *progressive* neurodegenerative disorder that unequivocally seems to have an environmental origin and a non-infectious etiology. This fatal disease is a complex clinical mixture of amyotrophic lateral sclerosis (ALS), parkinsonism (P) and dementia (D) with a neuropathology comparable to that of Alzheimer disease (AD) [3]. The affected brain accumulates many types of abnormal intracellular deposits (synuclein, *β*-amyloid, TDP-43), but it is dominated by deposits of microtubule-associated protein tau (MAPT), and therefore fits into the molecular neuropathological class of tauopathies [4]. The clinical and neuropathological features of this disease are best studied among Chamorro residents of the Mariana Islands of Guam and Rota [5] and among Japanese living in the Kii Peninsula of Honshu Island, Japan [6,7]. Least studied and without neuropathologic conformation is a third isolate of western Pacific ALS-PD complex (ALS-PDC) among Auyu and Jaqai linguistic groups in the southern lowlands of West Papua, on the Indonesian side of the island of New Guinea [8,9]. These three genetically distinct groups of people have experienced an extraordinarily high prevalence of this prototypical neurodegenerative disease, the latter flourishing in a setting in which no synthetic chemicals had ever ‘set foot’ at the time of its discovery. The same disease is non-transmissible from human to monkey, in contrast to kuru, a distinct but also disappearing neurodegenerative disorder formerly found among the South Fore people in the highlands of Papua New Guinea that is believed to have an infective prion etiology [10,11].

**Figure 1:**
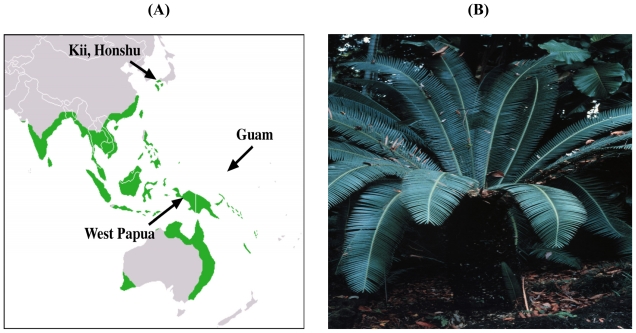
Cycad distribution in the western Pacific region (**A**) and photograph of a *Cycas circinalis* plant (**B**).

**Figure 2:**
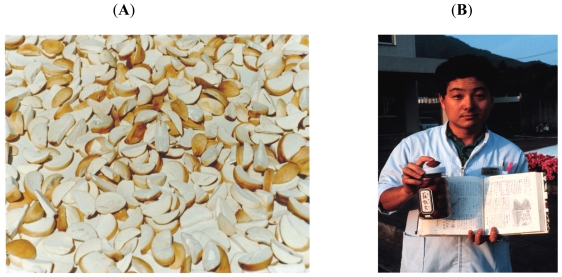
Cut seed of *Cycas micronesia*, a plant indigenous to Guam and the Micronesian islands, are processed for food by washing the halved or quartered seeds in water and laying them out to dry (**A**). Pharmacist in ALS-PDC focus in Kii Peninsula, Japan holding a labeled bottle of *Cycas revoluta* seed (*sotetsu*) and a textbook of folk medicine describing pharmaceutical indications for their medicinal usage (**B**).

The foregoing information suggests the operation of a naturally occurring environmental substance in the etiology of Western Pacific ALS-PDC. The only factor common to all three ALS-PDC foci is use for food and/or medicine of the seed of the hardy cycad plant (*Cycas* spp., Figures 1 and 2). Like all plant materials, the cycad seed is a complex mixture of chemicals, one of which (cycasin) is the principal subject of this paper. We hypothesize that the aglycone of cycasin, the potent genotoxin methylazoxymethanol (MAM), persistently perturbs cell signaling in the young adult brain because post-mitotic nerve cells are unable to repair MAM-induced DNA damage. We have solid evidence to support this conclusion for the developing rodent brain, which MAM readily disrupts, and the fingerprints of a comparable mode of action in the central nervous system of the young adult. If we are correct, MAM represents the first recognized agent with neurotoxic properties that produces a longlatency *progressive* neurodegenerative disease, where the interval between agent exposure and onset of neurological decline spans years or decades. We also present evidence that MAM perturbs cell-signaling pathways in the brain that are similar to those activated in mitotically-competent (cycling) epithelial cells that mutate and proliferate to form malignant tumors. This leads us to propose the possibility that cellular malignancy and progressive neurodegeneration are two sides of the same coin, the outcome depending on whether the genotoxin acts on a cycling or non-cycling cell, respectively. Since this idea deviates markedly from current understanding and has significant consequences for disease prevention and treatment, we hope our work will stimulate research on this question to prove or disprove our assertion.

We embark on this review cognizant of the incompleteness of the data we review in support of our hypothesis that unrepaired MAM-induced neuronal DNA damage perturbs cellular networks that may initiate neurodevelopmental delay in the immature brain or progressive neurodegeneration that begins in the young adult brain and appears clinically years or decades later in the form of the tauopathy ALS-PDC. Further, given the close neuropathological relationship between ALS-PDC and AD, we explore how new understanding of the former influences thinking about the possible role of environmental agents in the etiology of AD.

## 2. Evidence for the Role of Cycads in ALS-PDC

Since the etiology of western Pacific ALS-PDC is the subject of controversy, we should justify our position regarding the etiopathogenesis of this and other neurological diseases with which it has been compared. Several theories have been offered over the past 60+ years since high-incidence motorsystem disease was first recognized on Guam. Given its familial nature, inherited risk factors were first proposed, but with temporal decline in prevalence, some combination of genetic risk and environmental exposure was entertained. As disease rates continued to drop [12], the dominant clinical presentation changed over time from ALS to parkinsonism-dementia (PD) to Guam dementia (GD), and the age of onset continued to increase, it became increasingly clear that disease decline was most probably associated with a disappearing environmental exposure associated with the acculturation of Guam Chamorros to a western lifestyle. Screens of the Guam genome have failed to identify mutations responsible for Chamorro ALS-PDC but, in some cases, revealed polymorphisms in two independent *cis*-acting sites in the tau gene that might influence disease risk by regulating its expression [13]. Analysis of blood from Kii ALS-PDC subjects showed no mutations of 19 neurodegenerative disease-relevant genes [14]. Another finding in a subset of Guam ALS and PDC patients was a variant in the gene coding for the transient receptor potential cation channel, subfamily M, member 7 (TPRM7) [15], which produced functional channels with an increased sensitivity to inhibition by intracellular Mg^2+^ [16]. However, TPRM7 mutations have not been found in ALS-PDC cases in Kii peninsula [17]. ALS has also declined in the high-incidence disease focus of ALS-PDC in West Papua [9]. In sum, an inherited disorder is incompatible with the declining prevalence of ALS-PDC in all three geographic isolates, and no common causal genetic factor has been identified.

The longitudinal trend of ALS and PD, which has been documented epidemiologically for more than half a century on Guam, is consistent with an environmental agent that is declining hand-in-hand with post-World War II development of the island. Two hypotheses have been offered: one relates to declining use of the cycad plant for food and/or medicine [18], the other proposes that neurotoxic elements (such as aluminum and manganese) in water enter the body and are deposited in the brain. Low levels of calcium in water, it was suggested, triggered a parathyroid response that promoted gut absorption of calcium, with the result that absorption of “neurotoxic bystanders”, such as aluminum, would also take place [19]. While aluminum and other metals can be detected in the brain of Guam ALS-PDC patients, parathyroid function is unremarkable [20]. Additionally, in West Papua, ALS prevalence in a sessile, riverine population has declined without any known change in their source of drinking water [9].

The second environmental hypothesis focuses on exposures to neurotoxic components in seed of the cycad plant. The proposal originates from 1962 when the late Marjorie Whiting described the Chamorro practice of using cycad seeds for food and topical medicine [21]. Her research demonstrated that cycad plants had a long history in Australia and elsewhere of causing a neuromuscular disease in grazing animals. Cycads were found to harbor the first known plant mutagen, cycasin, and an uncommon amino acid, *β*-*N*-methylamino-L-alanine (L-BMAA), which induced an acute neurotoxic syndrome in chicks [22]. Interest in the cycad hypothesis lapsed until, decades after Whiting’s pioneering observations, we showed that primates treated orally with L-BMAA developed chronic degenerative changes in cortical and spinal motor neurons in association with a clinical picture reminiscent of the western Pacific disease [23]. However, because of the very high L-BMAA dosage used in this typical short-term toxicology experiment, and lack of disease progression after dosing ceased, the relevance of this model to ALS-PDC was subject to question. The cycad hypothesis again fell into disfavor until it was suggested the dosage of L-BMAA used in the earlier primate studies was realistic given that the Chamorro diet included flying foxes that harbored large concentrations of L-BMAA [24]. It should be noted, however, that flying foxes are not part of the diet of Japanese or New Guinean subjects at risk for ALS-PDC.

While Whiting emphasized the dietary use of cycads on Guam, the common exposure to cycad toxins in all three high-incidence disease foci is from *raw c*ycad seed used for medicine, with the additional use on Guam of the fresh seed cover to relieve thirst, the dried seed cover as a confection, and washed but incompletely detoxified cycad ovules as a source of flour for various food products [21] (Figure 2A). The neurotoxic cycad seed has been used as a topical treatment for skin lesions large and small [25], but such use undoubtedly declined as man-made pharmaceuticals were introduced. Oral medicinal use of cycad seed was also practiced in Japanese folk medicine in the Kii focus up through the 1980s, with prescriptions written by practitioners (*kitoshi*) that were filled by dispensing pharmacies (Figure 2B) [26]. Repeated oral or prolonged subcutaneous application of raw cycad seed was thus *common* to all three ALS-PDC isolates. The Chamorros of Guam and Rota also used the cycad seed ovule as a source of food. This was not remarkable since aboriginal groups in Australia historically prepared food from carefully detoxified cycad seed ovules without precipitating recognized neurological disease. Similarly, Japanese living in the Ryukyu Islands, where ALS-PDC is unknown, employed fermentation to eliminate cycasin [27]. Guam Chamorros, by contrast, used food preparation methods that only reduced the content of cycad toxins to “edible” levels that, while not lethal, nevertheless precipitated acute illness in children likely arising from the hepatotoxic properties of cycasin. We demonstrated residual levels of L-BMAA and cycasin in cycad flour prepared by families from different Guam villages [28].

### 2.1. Cycad Toxins: β-*N*-Methylamino-L-Alanine (L-BMAA)

L-BMAA is an uncommon amino acid that is synthesized by cyanobacteria and present in seed of *Cycas* spp. implicated in western Pacific ALS-PDC. Whether the amino acid is an endogenous cycad product or derived from invasive cyanobacteria is questioned. It should be noted that cyanobacteria also contain genotoxins that are capable of inducing DNA damage in both human and rodent cells [29]. We showed and others confirmed that L-BMAA is an excitotoxic amino acid with acute neurotoxic properties that are attenuated by glutamate receptor antagonists. Our group and several others have shown that L-BMAA has effects on both ionotropic and metabotropic glutamate receptors [30–33].

L-BOAA is a potent stereospecific amino acid in the Grass pea that acts in micromolar concentrations as an agonist of the α-amino-3-hydroxy-5-methyl-4-isoxazole propionic acid (AMPA) class of glutamate receptors. Although small concentrations of L-BOAA are present in cycad seed, lathyrism is a *self-limiting* pyramidal-tract disorder quite distinct from both the clinical form and progressive nature of western Pacific ALS-PDC. In a similar vein, domoic acid, which acts predominantly as a AMPA/kainate glutamate receptor agonist [36], induces a rapid-onset *self-limiting* neurodegenerative disorder [37]. Based on these observations, it is unlikely that the chronic motorsystem disease induced in primates fed L-BMAA arises from a membrane-based glutamate receptor action of the cycad amino acid. Stated otherwise, if L-BMAA does have a role in the etiology of western Pacific ALS-PDC, a mechanism other than glutamate excitotoxicity must be responsible. One possibility is described in the following paragraph.

**Figure 4:**
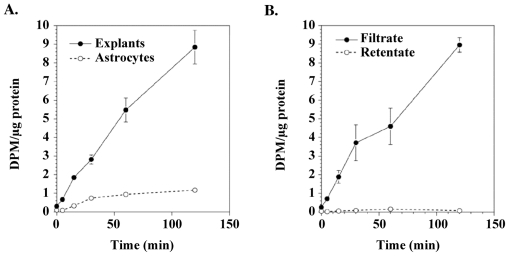

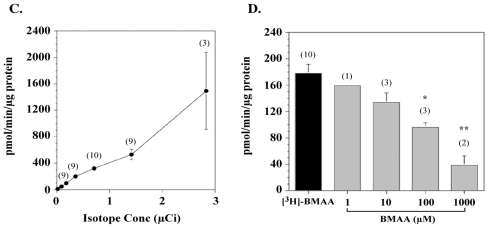
L-BMAA uptake into rat cortical explants, primary cortical astrocyte cultures and cortical synaptosomes after treatment with either L-[^14^C]-BMAA or L-[^3^H]-BMAA. (**A**) Uptake of L-^14^C-BMAA in mature mouse cortical explants and primary rat cortical astrocytes ^1^; (**B**) Distribution of L-[^14^C]-BMAA in mature mouse cortical explants treated with 1.6 mM of the neurotoxin ^2^; (**C**) L-[^3^H]-BMAA uptake into mouse cortical synaptosomes as a function of isotope concentration ^3^; (**D**) Effect of BMAA on L-[^3^H]-BMAA uptake into mouse cortical synaptosomes ^4^. Graphs derived from Kisby *et al.* [40] and unpublished data. ^1^ Explants from the motor cortex of 2-day old Swiss Albino mice were maintained for 2–3 weeks *in vitro* whereas cortical astrocytes derived from 1–3 day-old neonatal Sprague-Dawley rats were maintained for 3–4 weeks before treatment with 1.6 mM L-[^14^C]-BMAA (11.7 nCi/explant or culture) as previously described [40]. Note that the uptake of radiolabeled L-BMAA in mouse cortical explants was ~5-fold greater at 60 min than in comparatively treated rat cortical astrocytes. Values are mean ± standard error (n = 8–11 for explants and n = 6–7 for astrocytes) and corrected for non-specific binding of radiolabeled L-BMAA; ^2^ Explants were treated for various time periods (up to 2 h) with the radiolabeled neurotoxin (11.7 nCi/explant), washed with PBS (pH 7.4) and then homogenized in PBS. The tissue homogenate was ultrafiltered (10 K MWCO), washed with PBS and the radioactivity determined in the retentate (*bound*) and filtrate *(unbound or ‘free’*). Values are mean ± standard error (n = 4). Note that the radioactivity was found primarily in the filtrate; ^3^ Aliquots of L-[^3^H]-BMAA (17.88 μCi/mL buffer) were diluted with buffer and incubated for 5 min with crude synaptosomes as previously described [40]. Values are mean ± standard error. Number of determinations per concentration is shown in parentheses. ^4^ Synaptosomes were incubated for 5 min with radiolabeled neurotoxin (179 nCi) in the absence or presence of ‘cold’ BMAA (1.0 μM–1000 μM). Values are mean ± standard error. Number of determinations is shown in parentheses. Significantly different from treated synaptosomes incubated with L-[^3^H]-BMAA (* p < 0.05, ** p < 0.01).

L-BMAA might exert a chronic neurotoxic effect by being incorporated into protein, the foreign amino acid residue causing protein malfunction. The basis for this hypothesis comes from experimental evidence of significant levels of protein-associated L-BMAA in washed cycad flour and the detection of L-BMAA in the brain tissue of Guam Chamorros with ALS-PDC [38,39]. In support of this hypothesis, we showed that L-BMAA, but not l-BOAA, is taken up by cortical explants and synaptosomes in a time-dependent manner [40]. Previously unpublished data confirmed tissue uptake of L-BMAA in cortical explants treated with [^14^C]-L-BMAA and in mouse cortical synaptosomes treated with [^3^H]-L-BMAA (Figure 4). Additional data (*not shown*) demonstrated that L-BMAA is taken up and released from cortical synaptosomes. The rapid uptake, accumulation and release of L-BMAA, but not the structural analog L-BOAA, in cortical explants and synaptosomes suggest that the sub-chronic neurotoxic effects of this excitotoxin in primates is related to unknown intracellular actions.

Such a mechanism would be consistent with the slow evolution of motor neuron changes (chromatolysis) and the absence of excitotoxic damage in the frontal cortex of heavily dosed macaques exhibiting motorsystem (rather than convulsive) signs and the low levels of L-BMAA in the serum (micromolar) and cerebrospinal fluid (nanomolar) of one such animal [2,41]. Whether L-BMAA or its metabolite(s) target intracellular macromolecules critical for long-term maintenance of neuronal integrity is unknown.

**Figure 5:**
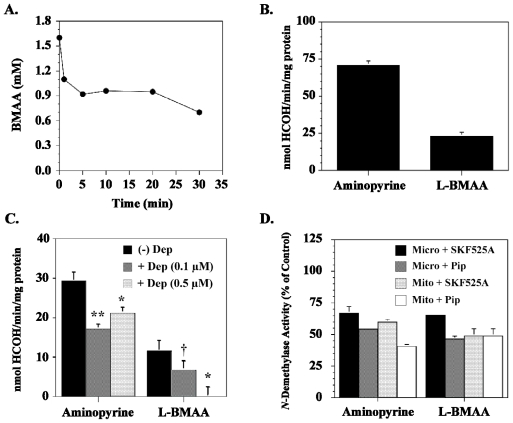
Metabolism of L-BMAA by the brain cytochrome P-450-dependent *N*-demethylase. (**A**) Disappearance of L-BMAA after incubation with rat cerebral microsomes ^1^; (**B**) Metabolism of L-BMAA by crude microsomes derived from mouse cortical explants ^2^; (**C**) Effect of the *N*-demethylase inhibitor deprenyl on the metabolism of L-BMAA by rat cerebral microsomes ^3^; (**D**) Effect of the cytochrome P450 inhibitors SKF525A and piperonyl butoxide on the metabolism of L-BMAA by rat cerebral microsomes and mitochondria ^4^. Graphs derived from Kisby *et al.* [43] and unpublished data. ^1^ Cerebral microsomes were prepared from adult Sprague-Dawley rats (n = 2) according to [44], diluted with buffer to a final concentration of 10 μg protein/mL and incubated at 37°C with 1.6 mM L-BMAA in the presence of 1.0 mM NADPH. At various time periods, an aliquot (100 μL) was ultrafiltered (10 K MWCO) and the ultrafiltrate analyzed for L-BMAA content by HPLC [45]. L-BMAA disappeared with time, an indication that it was metabolized by cerebral microsomes; ^2^ Explants (n = 15) were sonicated (2 × 10 s) on ice in 500 μl of ice-cold PBS, the homogenate centrifuged (16,000 × *g*) and an aliquot of the supernatant (crude microsomal fraction) taken for protein determination. The aliquot was incubated with 1.6 mM aminopyrine or L-BMAA and *N*-demethylase activity determined [44]. Samples were run in triplicate, averaged and the results expressed as nmoles of formaldehyde/min/mg protein ± standard error; ^3^ An aliquot of protein was incubated with 1.6 mM aminopryine or L-BMAA in the presence or absence of 0.1 μM or 0.5 μM deprenyl, an aminopyrine *N*-demethylase inhibitor [46], and *N*-demethylase activity determined [44]. Deprenyl had a significant influence on the metabolism of both aminopyrine and L-BMAA and the effect of this inhibitor on L-BMAA metabolism was concentration-dependent; ^4^ An aliquot of microsomal or mitochondrial protein was incubated with 1.6 mM aminopyrine or 1.6 mM L-BMAA in the presence or absence of either 100 μM SKF525A or 500 μMpiperonyl butoxide and *N*-demethylase activity determined [44]. Results are expressed as % *N*-demethylase activity relative to controls. The *N*-demethylase activity of control microsomes and mitochondria were comparable to that previously reported [44]. The *N*-demethylation of aminopyrine and L-BMAA was inhibited by piperonyl butoxide and SKF525A in both brain microsomes and mitochondria; an indication that L-BMAA is metabolized to formaldehyde by mixed function oxidases. Significantly different from microsomes incubated with aminopyrine or L-BMAA (*^†^* p < 0.05; * p < 0.01; ** p < 0.001).

L-BMAA is also metabolized to toxic species in rat tissue slices, mouse cortical explants, and rat crude cerebral microsomes. Nunn and Ponnusamy [42] reported that liver and kidney homogenates, but not brain homogenates, formed methylamine and 2,3-diaminopropanoic acid when incubated with L-BMAA. In contrast, Kisby and colleagues [43] found that rodent brain tissue metabolized both L-BMAA and aminopyrine (standard substrate for cytochrome P450) to the genotoxin formaldehyde, enzymatic reactions that were inhibited by the aminopyrine *N*-demethylase inhibitors deprenyl, SKF525A and piperonyl butoxide (Figure 5).

**Figure 6:**
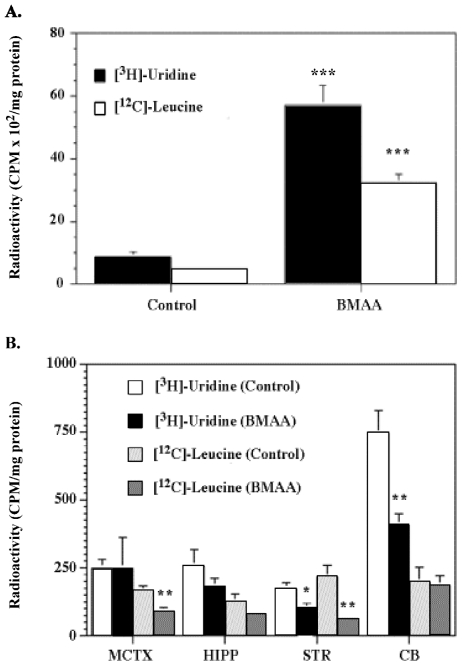
Effect of L-BMAA on rodent brain RNA and protein synthesis. (**A**) Effect of L-BMAA (24 h) on RNA and protein synthesis in mouse cortical explants ^1^; (**B**) Effect of L-BMAA on RNA and protein synthesis in various brain regions of adult rats ^2^. Graphs derived from unpublished data. ^1^ Explants from the motor cortex of 2-day-old Swiss Albino mice were maintained for 2–3 weeks before treatment for 24 h with 1.6 mM L-BMAA, as previously described by Kisby *et al.* [40]. Immediately after L-BMAA treatment, the explants were incubated with a solution containing [5-^3^H]-uridine (19.0 μCi) and L**-**[1-^14^C]-leucine (0.95 μCi), the explants washed with buffer, the tissue homogenate fractionated and the supernatant (RNA) and pellets (protein) examined for radioactivity as previously described by Pickard *et al.* [47]. Values are expressed as CPM/mg protein; ^2^ Adult male rats were administered a single intraperitoneal injection of vehicle (100 mM sodium bicarbonate, n = 8; Control) or L-BMAA (100 mg/kg, n = 8) daily for 14 days. Treated animals were injected intraperitoneally with a mixture containing [5-^3^H]-uridine (1.7 mCi/kg) and L**-**[1-^14^C]-leucine (84 μCi/kg); 30 min later, the brain was excised and dissected into various regions, homogenized and the tissue fractions examined for radioactivity as previously described [47]. Values are expressed as CPM/mg protein. Significantly different from control treated explants or brain tissue (* p < 0.05; ** p < 0.01; *** p < 0.001). MCTX = motor cortex. HIPP = hippocampus. STR = striatum. CB = cerebellum.

L-BMAA also interfered with brain RNA and protein synthesis when the amino acid was incubated with mouse cortical explants or administered intraperitoneally to adult rats (100 mg/kg) (Figure 6). In summary, while L-BMAA can act as a glutamate agonist, the amino acid is also taken up by brain tissue, forms a genotoxic metabolite, and regionally interferes with brain RNA and protein synthesis. These provocative findings suggest that further work on the non-excitotoxic properties of L-BMAA is merited, and it is premature to exclude the cycad amino acid as a participant in the etiology of western Pacific ALS-PDC.

### 2.2. Cycad Toxins: Azoxyglycosides and Methylazoxymethanol (MAM)

**Figure 7:**
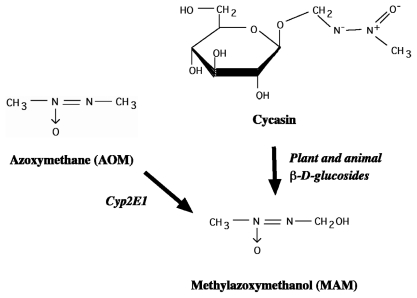
Structure of the cycad genotoxins cycasin (methylazoxymethanol-*β*-D-glucoside), methylazoxymethanol (MAM) and the related compound azoxymethane (AOM). The azoxyglycoside cycasin is converted to MAM by plant and animal *β*-glucosidases whereas azoxymethane is converted to MAM by mixed function oxidases (*i.e.*, P450 Cyp2E1).

While further study of L-BMAA and its genotoxic metabolite in relation to ALS-PDC is encouraged, the results of our recent toxicogenomic studies have focused attention on a possible and perhaps more important etiologic role for MAM (Figure 7), the genotoxic metabolite of cycasin. Whereas cycads contain small concentrations of L-BMAA, cycasin—the glucoside of the potent DNA alkylating agent MAM—accounts for 4% w/w of *Cycas* seed [48]. Cycasin is thus delivered in much larger doses than L-BMAA when the raw seed is used as an oral tonic (Kii, Japan) or for wound repair (Guam, West Papua). Secondly, there is a striking correlation between the cycasin content of cycad flour and the historical age-adjusted incidence of both ALS and PD among both male and female villagers of Guam [49,50]. Third, MAM acetate is a developmental neurotoxicant that is used by the experimental neurobiologist as a reliable tool to disrupt regional brain development. Cycasin also induces cycadism, a neuromuscular disorder seen in large grazing animals (cows, goats) after ingestion of cycads [51]. Cycadism can be triggered by cycad plants containing various azoxyglycosides other than cycasin, all of which release MAM upon enzymatic hydrolysis [48] (Figure 7).

However, experimental studies that have tested the effects of cycad and components thereof on laboratory species are difficult to interpret. An early study [52] reported the development of an ALS-like disorder (unilateral arm weakness) in one of three monkeys fed Chamorro-style cycad flour, but another cancer-focused study with a larger number of animals treated with cycasin or MAM neither reported the neurological status nor examined the nervous system microscopically [53,54]. Studies of adult rodents have focused on the carcinogenic potential of cycad and cycasin, although cerebellar pathology was reported in a study of rats fed L-BMAA [55]. A recent experimental study of rats fed cycad materials (cycasin content unknown) found neuropathological changes in the substantia nigra, striatum, locus coeruleus and cingulate cortex [56] which, as the authors note, supports the proposed role of cycad in western Pacific ALS-PDC, a proposal first made by Whiting and Kurland, later resurrected by Spencer and colleagues, and now once again the subject of intense biomedical interest. Shaw and his colleagues, the authors of the recent rodent study, propose that cycad sitosterols—either as the free sterol or the glucoside—are likely etiological agents; this seems unlikely since these compounds are used to treat prostatic hypertrophy without reported neurological disease, and controlled toxicological studies have given these sterols a clean bill of health [57].

## 3. Other Botanical Toxins, Toxicants and Neurodegenerative Disease

Western Pacific ALS-PDC has also been linked to a clinically similar atypical parkinsonism with tauopathy that occurs on the island of Guadeloupe in the Caribbean [58]. It is unclear whether this is a progressive disease; the initial report described self-limiting and even reversible forms of high-incidence parkinsonism and ALS. Guadeloupe tauopathy is associated with the heavy use for food and beverage of the sour sop or corossol (*Annona muricata*). It is claimed that Annonaceae have been commonly consumed in foci of atypical parkinsonism, including Guam, Guadeloupe and New Caledonia (where *Cycas* spp. also occur but uses in the latter two are unexplored). Annonaceae certainly merit further study for links with neurodegenerative disease because they contain substances with probable neurotoxic potential [59]. These include alkaloids and annonacin, the most abundant acetogenin, a potent mitochondrial Complex 1 inhibitor. Inhibitors of Complex 1 (1-methyl-4-phenyl-1,2,3,4 tetrahydro-pyridine, rotenone) or Complex 2 (3-nitropropionic acid) are linked with neurotoxicity, but generally precipitate *self-limiting* neurodegeneration in humans and laboratory animals. Cyanide-liberating cassava (*Manihot esculenta*), a plant with established neurotoxic potential, was also consumed on Guam, but not in Kii-Japan or West Papua. Dietary dependency on cassava in the absence of adequate protein intake can trigger a self-limiting neurodegenerative disease (cassavism) featured by spastic paraparesis which, like lathyrism, is quite distinct from western Pacific ALS-PDC [60]. Unlike the cycad, none of the above, notably cassava and sour sop, is common to all three foci of western Pacific ALS-PDC and neither induces a *progressive* motorsystem disease.

There are other clues implicating cycad seed in the etiology of western Pacific ALS-PDC, a disorder that affects systems other than the nervous system. Peripheral malignancy is an obvious potential outcome based on the mutagenic properties of cycasin and the compound’s ability to induce tumors in primates and rodents [53,54,61]. Unfortunately, there are sparse data on cancer incidence in Guam [62], and no attempt has been made to correlate tumor incidence with neurodegenerative disease or neurodevelopmental disorders. However, the integument and skeleton do provide possible links with cycadism. Ruminants grazing on cycad leaves lose their horns and hooves in the manner of a molt, where dermal regeneration is active [51]. In parallel fashion, cycad seed materials have been experimentally demonstrated in rodents to speed skin repair, which is consistent both with their therapeutic use to treat dermal injuries in Guam and West Papau, and with the resistance to bed sores of bedridden ALS patients on Guam (and elsewhere). ALS skin loses its normal elasticity and, on microscopic examination, is found to contain thin collagen and elastin fibers that are consistent with regeneration. Chamorros historically have had a high prevalence of diaphyseal aclasis, which is visible as non-malignant bony protuberances near the growing ends of long bones in the upper extremities [1]. In sum, these observations raise the possibility that a component within cycad activates molecular processes associated with epidermal growth. An unpublished study showing unusual skull bone thickness among Chamorros with ALS or PD is consistent with this idea.

## 4. Cycasin and Brain Development

“*Only small amounts of [cycad] are given to children because many become ill when they eat a dish made with cycad starch*” [21].

Given that cycasin is an established developmental neurotoxin, it would be of interest to understand if there is a relationship between cycad exposure during perinatal development and western Pacific ALS-PDC. Borenstein and colleagues [63] showed that exposure to cycads during childhood or young adulthood, but not adulthood, is an important risk factor for GD and PDC. Evidence of exposure to a genotoxin early in development is suggested by the presence of ectopic, multinucleated, Purkinje-like cells in the cerebellum of Guam and Kii subjects who died of ALS-PDC in middle or late life [64]. Guam ALS-PDC neurons with tau inclusions also contain mitotic markers [65]; such changes result from disruption of neuronal development, as occurs in neonatal rodents treated with cycasin or MAM [66]. These findings are consistent with emerging evidence indicating that neurons in AD and other tauopathies exhibit binucleation, cell cycle disturbances, and aneuploidy [67–69]. Thus, the work on the etiology of western Pacific ALS-PDC and related tauopathies has raised some interesting questions. Is the developing brain more vulnerable to cycad genotoxins (cycasin, MAM, L-BMAA?) than the mature brain? Do the early changes induced by cycad genotoxins in the developing brain herald the onset of changes that culminate in neurodegenerative disease? Similar questions were raised almost a decade ago to explain how environmental factors might elicit progressive neurodegenerative disorders such as Parkinson’s disease (PD) and AD [70]. Answers to these questions are likely to provide further insight into the underlying mechanisms of GD, PDC and related tauopathies, since there is growing evidence that neurodegeneration and carcinogenesis share a number of cellular pathways [71,72].

**Figure 8:**
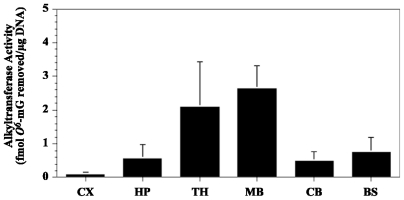
*O^6^*-Methylguanine methyltransferase (MGMT) activity in various brain regions of neonatal mice. Protein extracts (100 μg) from postnatal (6–8) brain tissue of wild type (C57BL6) mice (n = 3) were incubated with [^3^H]-methyl-DNA and the amount of [^3^H] *O^6^*-methylguanine determined by HPLC with liquid scintillation counting. Values are the mean ± standard error. CX = cortex, HP = hippocampus, TH = thalamus, MB = midbrain, CB = cerebellum, BS = brainstem (graph of unpublished data kindly provided by S.L. Gerson, Case Western Reserve University, Cleveland, OH, USA).

We have explored the relationship between early life exposure to cycasin and western Pacific ALS-PDC by examining the response of the developing rodent brain to MAM, the active metabolite of cycasin. MAM alkylates brain tissue DNA to produce N7-methylguanine (N7-mG) and *O^6^*-methylguanine (*O^6^*-mG) DNA lesions [48] when administered to rodents either *in utero* [73,74] or after birth [75,76]. N7-mG and *O^6^*-mG DNA lesions are repaired by the base-excision and direct reversal (*i.e.*, *O^6^*-methylguanine methyltransferase, MGMT) repair pathways, respectively [77]. These DNA lesions also accumulate and may persist in the brain of fetal rats or neonatal mice after a single injection of MAM or related alkylating agents [48,75,78] because the repair of these DNA lesions in the rodent brain varies across different regions (Figure 8) and is significantly less efficient than in other organs (e.g., liver) [79]. *O*^6^-mG DNA lesions have greater persistence in rodent brains [48,75,78], and they are highly mutagenic at the level of transcription in human cells, leading to an altered protein load, especially in cells with significantly reduced MGMT [80]. Since the fetal and young adult human brain also have a low or absent capacity to repair alkylation-induced DNA damage [81,82], the immature rodent is a good animal model to examine the response of the human brain to the cycad genotoxin MAM.

Global gene expression profiling [75] and proteomic studies [83] of the brain of neonatal mice administered a single injection of MAM (21.5 mg/kg) showed that the cycad genotoxin produces an early and persistent increase in DNA damage, and disrupts the expression of genes and proteins that regulate the neuronal cytoskeleton, protein degradation and mitochondrial metabolism. Global gene expression profiling of young post-mitotic cerebellar neurons and astrocytes treated *in vitro* with MAM demonstrated that immature neurons are more vulnerable than astrocytes, and this vulnerability to MAM is associated with the accumulation of DNA lesions and distinct alterations in gene expression [84]. The preferential targeting of genes involved in such diverse functions as cell signaling, transcriptional regulation, differentiation, and the stress and immune response, suggests that MAM targets distinct neuronal networks in the developing brain. This is supported by the insensitivity of adult neurons to MAM [66]. Since these cellular pathways are also disturbed in neurodegenerative disease, and the undeveloped human brain is particularly inefficient at repairing alkyl DNA lesions (*i.e.*, *O^6^*-mG) [81,82], MAM may have targeted one or more of these pathways by a DNA damagemediated mechanism. While *O^6^*-mG DNA lesions are cytotoxic and mutagenic (in cycling cells), their persistence for long periods in neurons [78] could disrupt brain function and provide the critical substrate for enhanced predisposition to late-life neurodegenerative disorders. Therefore, the DNA damage produced by MAM in the undeveloped human brain may constitute an important, but relatively unacknowledged cause of the synuclein and tau pathology in western Pacific ALS-PDC and other tauopathies [63,85].

While DNA damage is a characteristic feature of synucleinopathies and tauopathies [86–89], how the DNA damage produced by cycad genotoxins might contribute to western Pacific ALS-PDC is not understood. To address this central issue, we compared the response of the neonatal brain of DNA repair-proficient (wild type, C57BL6/J), –deficient (*Mgmt^−/−^*) and – overexpressing (*Mgmt^Tg+^*) mice to MAM or related genotoxins including the methylating agent dimethyl sulfate (DMS), the monofunctional or bifunctional chloroethylating agents chloroethylamine (CEA) and nitrogen mustard (HN2) [75,76]. MAM methylates DNA to produce N7-mG and *O^6^*-mG DNA lesions, whereas HN2 rapidly and irreversibly alkylates guanine (G) and adenine (A) (e.g., N7-alkylG and 3-alkylA, respectively) of DNA to produce monoadducts, intrastrand cross-links and interstrand cross-links. Dimethyl sulfate is a methylating agent that produces primarily N7-mG and 3-methyladenine DNA lesions, while CEA, the monofunctional analogue of nitrogen mustard, produces predominantly N7-alkylG DNA lesions, but not cross-links. The primary objective of using MAM and related alkylating agents was to determine if these genotoxins induce their long-term effect on neurons in the developing brain through the generation of specific DNA lesions. By comparing the response of the neonatal brain to MAM and related alkylating agents among these DNA repair genotypes, we would be able to determine if the DNA lesions produced by the cycad genotoxin are linked to neuropathological and associated neurobehavioral changes. Neurodevelopment and motor function were more severely affected by MAM in *Mgmt^−/−^* mice than after treatment with other alkylating agents (*i.e.*, dimethylsulfate, nitrogen mustard), and these effects were less pronounced in similarly treated wild-type mice [76]. The reduced number of *Mgmt^Tg+^* neurons undergoing apoptosis and the preservation of cerebellar morphology and motor function in MAM-treated *Mgmt^Tg+^* mice is additional evidence that MGMT plays an important role in protecting the undeveloped brain from cycad genotoxin-induced injury. These findings indicate that one pathway by which the cycad genotoxin MAM induces its neurotoxic effects is through the production of *O^6^*-mG DNA lesions.

## 5. Cycasin and the Young Adult Brain

Estimates suggest that exposure of *young adults* to the picking, processing and eating of cycad food products (*fadang*) incurred the highest attributable risk for mild cognitive impairment (MCI), GD and PDC among Guam Chamorros [63,85]. In comparison, there was no association between the picking, processing or eating of *fadang* among *adults* and risk for MCI, GD, or PDC. Thus, the incompletely developed human brain appears to be especially vulnerable to cycad genotoxins. We explored this hypothesis further by examining the response of the young adult murine brain to the cycad genotoxin MAM. As in the neonatal studies [75,76], both DNA repair-proficient and *Mgmt* knock-out (*Mgmt^−/−^*) mice were used to determine if DNA damage also plays an important role in the response of the young adult brain to cycad genotoxins.

Global gene expression profiling was used to compare the extent of MAM-induced DNA damage (*i.e.*, *O^6^*-mG) with the response of the young adult brain of both DNA repair-proficient mice (C57BL6/J) and DNA repair-deficient (*Mgmt^−/−^*) animals [90]. The brains of young adult *Mgmt^−/−^* mice treated with a single systemic dose of MAM showed significantly higher levels of *O*^6^- mG than the brains of comparably treated C57BL6/J wild-type mice. The DNA damage in the brain of MAM-treated young adult *Mgmt^−/−^* mice remained elevated (up to 7 days post-treatment), an indication that *O*^6^-mG lesions are persistent and MGMT is essential for removing the DNA damage (whether produced by MAM or related alkylating agents) [78,91,92]. The *O*^6^-mG levels were linked to changes in the expression of genes in several cell-signaling pathways (*i.e.*, TP53, NF-kB, MAPK) associated with cancer, neurological disease, neurodevelopmental and skin disorders. These data are consistent with the established developmental neurotoxic and carcinogenic properties of MAM in rodents [93–96]. The prominent modulation of ‘cancer genes’ in the “tumor-insensitive” brains of MAM-treated young adult animals suggests that perturbations of these genes in the undeveloped brain have consequences other than cancer. They also support the hypothesis that early-life exposure to MAM-glucoside (cycasin) has an etiological association with western Pacific ALS-PDC.

The experience of Chamorro migrants from Guam to the continental United States shows that ALS-PDC may surface clinically years or decades after they leave the Guam environment [97]. Similarly, occasional non-Caucasian migrants to Guam have lived in the Chamorro culture for years or decades before developing clinical signs of ALS-PDC [98]. Comparable “latent periods” have been noted in individuals who developed ALS after treatment with raw cycad seed either by the oral route (Kii-Japan) or subcutaneously (West Papua) [25,26]. Neuropathological signs of subclinical tauopathy have been reported in Chamorros who died without evidence of neurological disease [99]. Taken, in concert, therefore, the agent responsible for the induction of western Pacific ALS-PDC triggers a pathological process that advances long after exposure has terminated. We have therefore undertaken preliminary studies to determine if MAM induces latent transcriptional changes in the brains of *Mgmt^−/−^* *vs.* wild-type mice. Brain transcriptional changes 6 months after single systemic treatment with MAM (MAM_late_) were comparable to those seen at 7 days post-MAM treatment, and there was elevation of mitogen-activated protein kinases and increased caspase-3 activity, both of which are involved in tau aggregation and neurofibrillary tangle formation typical of ALS-PDC and AD [100]. Additionally, the MAM_late_ transcriptional profile was dominated by the presence of 28 genes involved in olfactory transduction, which suggests the presence of a MAM-induced change in olfaction status. While caution is merited when comparing rodent and human data, olfactory dysfunction is among the first signs of neurodegenerative disease [101]. Marked olfactory deficits, first reported in Guam PDC, are also similarly present in Chamorro patients with ALS, pure parkinsonism, and pure dementia, and in some controls with possible sub-clinical ALS-PDC [102]. Olfactory deficits are also among the first signs of AD and idiopathic PD [103,104]. The inability to distinguish the nature of olfactory dysfunction among Guam PDC, AD [105] and ALS patients [102], suggests a common neurologic substrate and underlines the close relationship between ALS-PDC and the more familiar neurodegenerative disorders seen in the West.

These findings suggest that environmental genotoxins, specifically MAM, target common pathways involved in neurodegeneration and cancer, the outcome depending on whether the cell can divide (cancer) or not (neurodegeneration) [90]. Both phenotypes, it should be noted, represent diseases that surface clinically long after exposure terminates. Others have proposed links between neurodegeneration/cancer and cell cycle regulation, DNA repair, response to oxidative stress [72,106], aberrant wingless and proto-oncogene Int-1 (Wnt) signaling [107], glycogen synthase kinase 3-*beta* (GSK3β) regulation [108], modulation of tumor protein 53 (TP53 or P53) expression [109], and perturbations of tau in AD and prostate cancer [110]. Chronic tissue inflammation is another characteristic feature of both cancer, AD [111] and western Pacific ALS-PDC brain. Further investigation of the cell signaling pathways that are activated by MAM in the immature brain might help us understand how the disruption of ‘cancer genes’ could contribute to neurodegenerative disease or neurodevelopmental disorders.

**Figure 9:**
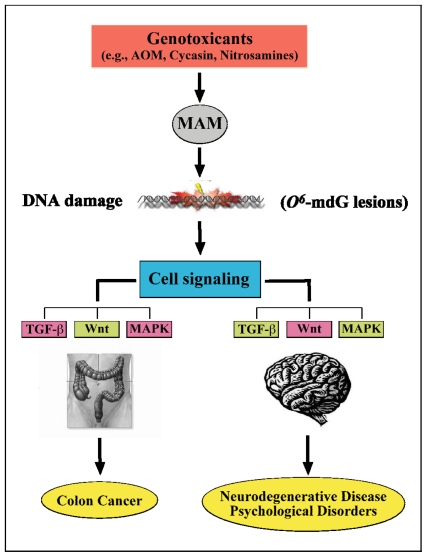
Proposed relationship between MAM-induced colon cancer and brain disease/disorders. Genotoxins, such as methylazoxymethanol (MAM), that induce *O^6^*-methyldeoxyguanine lesions (*O^6^*-mdG DNA damage), disturb cell signaling pathways, including transforming growth factor-β (TGF-β), wingless and proto-oncogene Int-1 (Wnt), and mitogen-activated protein kinase (MAPK). In general, the literature supports up-regulation (green) and down-regulation (pink) in association with the two distinct phenotypes. (Modified from Chen and Huang [112]). Thus, MAM may have opposing effects that depend on the mitotic state of the cell: whereas the agent may induce mutagenesis and proliferation (tumorgenesis) in cycling cells, in non-cycling cells (*i.e.*, neurons) the outcome is cellular degeneration.

These provocative findings suggest that the cycad genotoxins cycasin and MAM induce their neurotoxic effects through molecular mechanisms similar to those reported in the azoxymethane (AOM) mouse model of colorectal adenocarcinoma in which MAM (the cytochrome P450 2E1 mediated metabolite of AOM; see Figure 7) is the sole triggering agent [96,112]. In the AOM mouse model, MAM-induced *O^6^*-mG DNA lesions lead to mutations in *K*-ras (*i.e.*, transversion from G:C to A:T at codon 12) and β-catenin (*i.e.*, transversion from G:C to A:T at codons 33, 34, 37, and 41; serine and threonine sites phosphorylated by GSK-3β) [113,114], resulting in the downstream activation of several cell signaling pathways, including phosphoinositide 3-kinase/Akt (PI3K/Akt), MAPK and Wnt [112]. Such events may explain how MAM modulates the expression of genes with pivotal roles in cell signaling in the brain of young adult mice (Figure 9).

**Figure 10:**
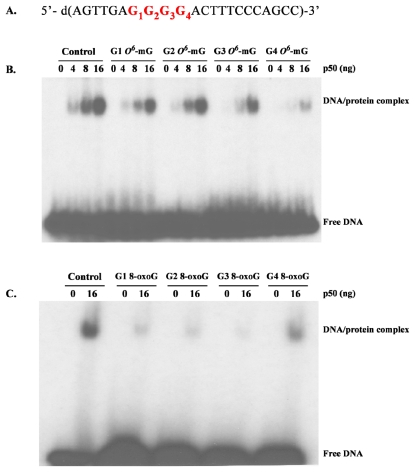
Autoradiograms showing the gel shift induced by binding of the p50 subunit of NF-kB to its DNA recognition sequence containing a DNA lesion. (**A**) Recognition sequence for the p50 subunit of NF-kB. Four oligonucleotides, each containing a guanine (G) or either an *O^6^*-mG (**B**) or 8-oxoG (**C**) located in the consensus sequence of the p50 subunit, were incubated with different concentrations of purified p50, electrophoresed and the autoradiograms examined for bands by chemiluminescence. The concentration dependence of the DNA-p50 complex was demonstrated by the addition of 0, 4, 8, or 16 ng of purified p50 in the reaction mixture. This suggests that binding of the transcription factor (NF-kB) to its cognate DNA recognition sequence is altered both by the nature and the specific location of the DNA lesion (images derived from unpublished data).

Since neurons in the young adult brain are post-mitotic, MAM-induced *O^6^*-mG DNA lesions likely perturb these pathways by disrupting the binding of transcription factors [115–119] or by activating transcriptional mutagenesis [80]. This is supported by recent studies showing that the binding of the p50 subunit of the NFkB transcription factor, a cell-signaling pathway that is perturbed by MAM in the rodent brain [90], is altered by a single *O^6^*-mG or 8-oxoG DNA lesion (Figure 10).

The location as well as the type of DNA damage had a pronounced influence on binding indicating that a persistent DNA lesion could have a significant effect on gene expression. However, if these DNA lesions occur in the transcribed strand of an active gene, they could miscode leading to mutant transcripts and proteins, a process known as transcriptional mutagenesis [120,121]. Recent studies by Burns and colleagues [80] provide strong support for this hypothesis by showing that a single *O^6^*-mG DNA lesion located within the coding sequence of a reporter gene produced both altered RNA and protein levels. An active gene that contains *O^6^-*mG in a position sensitive to T or C transition could produce as much as 66% altered transcripts. If these lesions persist in the young adult brain, they would have a detrimental influence on brain function.

## 6. Relevance to Neurodegenerative Disorders

While the proposed link between human exposure to cycad genotoxins (cycasin, MAM, L-BMAA?) and western Pacific ALS-PDC is controversial, equally provocative suggestions have raised the possibility that related chemicals in the North American environment may be linked to sporadic PD and AD. Epidemiological studies published a couple of years ago by de La Monte and colleagues [122] noted strong parallels between the age-adjusted increases in the death rate from AD, PD and diabetes mellitus, and progressive increases in human exposure to nitrates, nitrites and nitrosamines through processed and preserved foods as well as fertilizers. By contrast, other diseases, including HIV-AIDS, cerebrovascular disease, and leukemia, did not exhibit those trends. De la Monte and colleagues propose that the increase in exposure to these environmental chemicals plays a critical role in the cause, development and the effects of these diseases through an insulin-resistant mechanism. Just as the cancer research community decades ago called for a reduction in exposure to nitrite preservatives in food [123], the question of a relationship between exposure to such compounds and neurodegenerative disease is now an issue. The basis for focusing on nitrite preservatives is their transformation in the gut to genotoxic nitrosamines.

*N*-Nitrosamines are potent carcinogens that occur widely in the environment, and they are also formed endogenously in the stomach following the interaction of ingested nitrate or nitrite with secondary amines (e.g., proteins) [124]. Human exposure to nitrosamines or their precursors, nitrates and nitrites, can also occur from exogenous sources, such as diet [125], drinking water [126], occupation (e.g., rubber industry) [127], or environmental exposures (Table 1). Nitrosamines, like MAM, are potent alkylating agents that methylate DNA to produce N7-mG and *O^6^*-mG DNA lesions [128]. The antineoplastic agent streptozotocin (STZ), a monofunctional nitrosourea derivative isolated from *Streptomyces achromogenes*, is also a potent alkylating and highly genotoxic agent known to directly methylate DNA. Since antineoplastic agents impair cognition [129] and the effects are persistent [130], these agents might also induce both structural and functional changes in the immature brain through a DNA damage-mediated mechanism.

**Figure 11:**
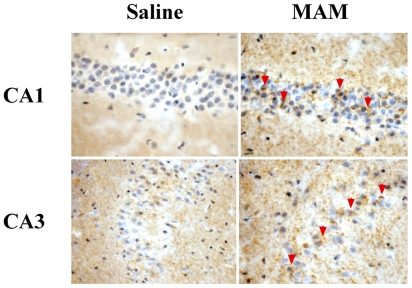
MAM induces Tau phosphorylation in the brain of *htau* mice. Neonatal (PND3) *htau* mice (mouse mutant that overexpresses normal human Tau) were given a single subcutaneous injection of saline or MAM (22 mg/kg). The hippocampus of the offspring was examined at post-natal day 90 for the phosphorylation of Tau at serine 199 (pS199). Note the heavy accumulation of pS199 within the axons (*arrowheads*) of CA1 and CA3 pyramidal neurons after MAM treatment. Tissue slides were prepared for immunohistochemistry according to Kisby *et al.* [83]. Magnification, x40 [images kindly provided by R. Woltjer (OHSU) and derived from unpublished data].

MAM reproducibly induces brain maldevelopment by a DNA damage mechanism [76], but how this cycad toxin might induce chronic neurodegenerative disease is not understood. One possibility is that MAM perturbs the brain insulin-signaling pathway like the related genotoxins STZ [132] and nitrosamines [133]. STZ, a glucosamine of *N*-methylnitrososurea, induces a rodent model of dementia that is characterized by progressive deterioration of memory, energy metabolism [134], and tau pathology [135,136]. The tau pathology in the STZ rodent model of dementia is preceded by early changes in the phosphatidylinositol-3-kinase (*i.e.*, PI3K, phospho-Akt, GSK-3β) insulin/IGF-signaling pathway [137]. Nitrosamines also perturb the brain insulin/IGF-signaling pathway (*i.e.*, insulin, IGF, IGF receptors, GSK 3β) when administered to neonatal (postnatal day 3) rats [133,138]. Like STZ, the effect of nitrosamine on the brain insulin/IGF-signaling pathway precedes the tau pathology (*i.e.*, tau, phospho-tau) and cognitive impairment. MAM induces similar changes in the brain of mice that overexpress normal human tau *(i.e.*, htau) (Figure 11) suggesting this environmental genotoxin is also capable of inducing tau pathology by a mechanism similar to that activated by streptozotocin and nitrosamines. Since IGF and GSK-3β are also perturbed in the brain of Guam and Japanese ALS/PDC patients [139], and insulin resistance is an established risk factor for dementia [140,141], the cycad genotoxins might induce tau pathology in GD and PDC by inducing brain insulin resistance.

## 7. Summary and Putative Molecular Mechanisms

Western Pacific ALS-PDC, a neurodegenerative disease prototypical for human tauopathies such as AD, has been described in three geographic isolates among genetically distinct people. The common features of the disease in Guam, Kii and West Papua are the combination of high-incidence clinical ALS, PD and overlapping forms in single populations; decreasing overall prevalence of ALS-PDC with societal development, with ALS declining before PD; and a common neuropathology (not studied in West Papua). The cause of ALS-PDC is unknown but a common, non-infectious environmental factor with declining access to the three high-risk groups is highly probable. The leading candidate for the culpable agent is one or more neurotoxins in cycads, ancient plants long established as causal of neuromuscular disease in grazing animals. Raw cycad seed has been used for medicinal purposes in all three ALS-PDC foci, and incompletely detoxified cycad seed flour has been an important food item in Guam, as have cycad-eating flying foxes: these traditional practices have declined with acculturation to modern life. Cycad chemicals include (among many others, most of which have yet to be identified) the uncommon amino acid L-BMAA, a compound with excitotoxic properties mediated by glutamate receptors and the azoxyglucoside cycasin, which forms the potent developmental neurotoxin MAM. Both MAM and L-BMAA are taken up by brain tissue, where they are metabolized to compounds with genotoxic properties including the carcinogen formaldehyde, which has twice been linked with ALS in the US and UK [142,143]. Primates fed large doses of L-BMAA develop a motorsystem disease featured by chronic degenerative changes of cortical and spinal motor neurons, neurofibrillary tangles, and extrapyramidal dysfunction. MAM induces DNA lesions that markedly disrupt rodent brain development and, in the young adult brain, activate intracellular signaling pathways linked with human neurological disease (tauopathies) and cancer. Neurodegeneration and cancer may be associated with similar pathways in cycling cells and non-cycling cells (neurons), respectively, the ability of the cell to divide, rather than an intrinsic toxic property of the culpable agent, determining phenotypic outcome. Labeling MAM and comparably acting chemicals as “carcinogens” or “neurotoxins” is therefore misleading. MAM-like alkylating agents (nitrosamines, hydrazine, streptozotocin), which are known for their carcinogenic potential, should be examined for potential long-latency neurotoxicity.

If one or more cycad genotoxins play a role in the etiology of western Pacific ALS-PDC, we must explain both the differential clinical presentation (ALS, PD, GD) and the long latent period (years, decades) that intervenes between exposure and the appearance of clinical disease. Since ALS relative to PD usually occurs in younger patients, the time to onset of the motor neuron disease ALS is relatively short. This might result from early-life exposure to cycad materials or heavy exposure(s) to the culpable toxin, either or both precipitating clinical disease at younger ages. Since early-life exposure to cycads has been linked to Guam PD and GD, disorders that have a mean age of onset substantially higher than that of Guam ALS, this suggests that dosage is a critical factor in determining clinical outcome. Viewed from this perspective, therefore, large exposures may trigger fatal motor neuron disease (ALS and sub-clinical parkinsonism) in the relatively young (> 20 years of age), whereas lower exposures affect motor neurons to a lesser degree and thereby allow the subject to survive to an older age whereupon they develop PD or, later in life, Alzheimer-like Guam dementia.

Another crucial question is the biological mechanism by which a cycad genotoxin can act as a “slow (neuro)toxin”, that is an agent that (like a carcinogen) triggers a neurodegenerative disease process that is initially covert but which, over a sufficient period of time, surfaces in clinical disease and progresses to a fatal outcome. Among the various possibilities, four pathogenic mechanisms may be considered: all postulate a persistent change in nerve cells, either as a result of unrepaired DNA damage or the presence of a mutant protein.

One possibility postulates that normal synaptic activity, perhaps involving glutamate metabotropic receptors, would continuously drive DNA-damaged neurons to activate signal pathways involved in tau regulation, resulting in abnormal tau production, tau-rich neurofibrillary tangles and, ultimately, neuronal degeneration. Neurofibrillary brain pathology would be evident in sub-clinical forms of the disease but, as neuronal degeneration advanced relentlessly, the disease would eventually manifest clinically. With the Hugon laboratory, we tested the biological principle underlying this hypothesis using primary cultures of rat cortical neurons that responded to a short (“synaptic-like”) pulse of exogenous glutamate with a measurable increase in *tau* transcription. A comparable pulse of DNA-damaging MAM also increased tau mRNA, but when the application of MAM preceded that of glutamate, the resulting increase in *tau* transcription was greater than the sum of the two in isolation [144,145].

A second possibility is a mechanism known as transcriptional mutagenesis, which involves a persistent lesion in the coding region of a gene. While the mutagenic consequences of exposure to DNA-damaging agents like MAM can trigger cell proliferation in cycling cells (such as gut cells) leading to tumor formation, the consequences for transcription in non-cycling cells (such as neurons) are unknown, but perhaps include degeneration. Studies performed by Bregeon and colleagues [120] examined transcriptional activity on a DNA template containing a DNA lesion (8-oxoguanine) placed at defined positions along the transcribed strand of a reporter gene. Luciferase expression was efficient, but error-prone transcriptional by-pass of 8-oxoguanine occurred *in vivo*, and this lesion was not repaired by the transcription-coupled repair machinery of mammalian cells. “Analysis of luciferase expression from 8OG:C-containing constructs showed that the generated aberrant mRNAs led to the production of mutant proteins with the potential to induce a long-term phenotypical change. These findings reveal that erroneous transcription over DNA lesions may induce phenotypical changes with the potential to alter the fate of non-replicating cells.” [120].

The third possibility is another form of transcriptional disruption associated with persistent lesions in the promoter region of a gene leading to erroneous expression of the associated protein. Bonfanti and colleagues [115] showed that *O^6^*-mG, one of the DNA lesions produced by MAM, inhibited the binding of transcription factors to their cognate DNA sequences in a position-dependent manner. The *O^6^*-mG lesion blocked DNA binding of NF-kB, Sp1 and SRF. We confirmed that binding of NF-kB to its cognate sequence can be disrupted in a position-dependent manner by the presence of *O^6^*-mG in DNA (see Figure 10). The importance of these observations is that DNA lesions within the promoter (*vs.* the coding) regions of genes may produce persistent up- or down-regulation of genes. This may explain why NF-kB was a prominent hub in the brains of MAM_early_ and MAM_late_ animals.

A fourth possibility that involves L-BMAA specifically is the substitution of this plant amino acid for alanine in protein synthesis, another route by which mutant proteins could be produced. Protein-bound L-BMAA has been reported in cycad flour and in brain tissue from Chamorro subjects with ALS-PDC [38,146]. Mutant neuroproteins are established causes of progressive human neurodegenerative diseases, such as kuru and other fatal disorders involving mutations (misfolded) neuronal prion proteins [147]. Kuru is a subacute neurodegenerative disease with tau-containing plaques; there is heavy involvement of the cerebellum as well as the caudate, putamen, and much of the cerebral cortex [148]. While the infectious property of the mutant prion protein has been the subject of great public health interest, there is no research on the gene-based mutational event that presumably triggered production of the first mutant prion protein. In this regard, it is noteworthy that the kuru-affected South Fore people of Papua New Guinea were exposed to cycad mutagens through the practice of chewing the fleshy cycad seed cover and spitting the contents into the food of kuru victims. Indeed, in the West Papua focus of ALS-PDC, where the ovule is only used as topical medicine for large open wounds, cycad seed is named “kurru”, meaning, simply, “seed of the tree” [9]. Is there an undiscovered link between kuru and the developmental neurotoxicity of MAM, which produces prominent cerebellar or cortical pathology depending on the timing of application?

## Figures and Tables

**Figure 3:**
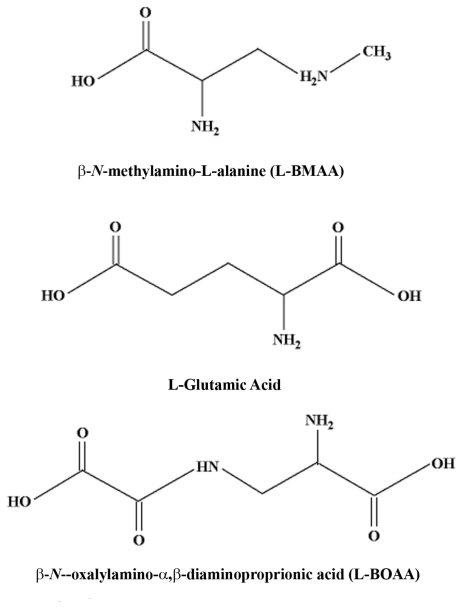
Structures of the amino acid neurotransmitter L-glutamate and the excitotoxic amino acids *β*-*N*-methylamino-L-alanine (L-BMAA) and *β*–*N*-oxalylamino-L-alanine (L-BOAA). Note the structural similarity between glutamate and both L-BMAA and L-BOAA.

**Table 1:** Common Sources of *N*-Nitrosamines.

Source	*N*-Nitrosamine †	Concentration
*Food/Beverages*		
Bacon	DMN, DEN, NPYR	1–40 ppb
Salami	DMN	10–80 ppb
Luncheon meat	DMN, DEN	1–4 ppb
Hamburger	DMN	15–25 ppb
Chinese marine salted fish	DMN DEN	0.05–21 ppm
Smoked salmon	DMN	0–26 ppb
Cheese	DMN	1–4 ppb
Wheat flour	DEN	0–10 ppb
Beer	DMA	

*Commercial Products*		
Latex gloves	DMN	37–329 ppb
	DEN	< 10 μg/kg
Cigarettes	DMN, DEN, NPYR	0–180 ng/cig.
Pesticide Formulations	DMN	300 ppb–640 ppm
Rubber nipples	DMN, DEN	< 100–200 μg/kg
Rubber toys	DMN	< 25 μg/kg
Rubber balloons	DMN	< 150 μg/kg
	DEN	> 30 μg/kg

*Industrial Exposure*		
Leather Tanneries	DMN	0.1–47 μg/m^3^
Foundries, USA	DMN	0.062–2.4 μg/m^3^
Rubber, curing, salt bath	DMN, DEN	
Rubber, curing, injection molding	DMN	

* Source: Thomson *et al.* [131];

† Abbreviations: DMN (NDMA): *N*-nitrosodimethylamine; DEN (NDEA): *N*-nitrosodiethyl- amine; NPYR: *N*-nitrosopyrrolidine; DMA: *N*-nitrosodimethylamine.
